# Efficacy of inverted internal limiting membrane flap for treating optic disc pit maculopathy

**DOI:** 10.1093/jscr/rjaf678

**Published:** 2025-08-29

**Authors:** Anny M S Cheng, Alfredo A Paredes, Kakarla V Chalam

**Affiliations:** Department of Ophthalmology, University of Florida, 6201 W Newberry Rd, Gainesville, FL 32605, United States; Department of Ophthalmology, Florida International University, Herbert Wertheim College of Medicine, 11200 SW 8th St AHC2, Miami, FL 33199, United States; Department of Medicine, Florida Atlantic University Charles E. Schmidt College of Medicine, 777 Glades Road BC-71, Boca Raton, FL 33431, United States; Department of Ophthalmology, Loma Linda University, 11370 Anderson St., Suite 1800, Loma Linda, CA 92354, United States

**Keywords:** central foveal thickness, inverted internal limiting membrane flap, optic disc pit maculopathy, pars plana vitrectomy

## Abstract

Optic disc pit maculopathy is a serious complication of congenital optic disc pits, often resulting in significant vision loss due to intraretinal and subretinal fluid accumulation. This case report describes a 35-year-old woman with progressive visual decline in her right eye. Examination revealed optic disc pit maculopathy with marked central foveal thickness (634 μm) and a best-corrected visual acuity of 20/100. The patient underwent pars plana vitrectomy, induction of posterior vitreous detachment, selective internal limiting membrane peeling to create a temporal flap, inversion of the flap over the optic disc pit, and gas tamponade with 25% sulfur hexafluoride. At 8 months postoperatively, there was complete resolution of retinal fluid, reduction in central foveal thickness to 261 μm, and best-corrected visual acuity improved to 20/20. These anatomical and functional gains were maintained at 16 months, highlighting the efficacy and safety of the inverted ILM flap technique in optic disc pit maculopathy management.

## Introduction

Optic disc pit (ODP) is an uncommon, congenital anomaly of the optic nerve head, typically presenting as an oval, gray-white depression located at the inferotemporal aspect of the disc [1]. Although ODPs are frequently asymptomatic, they may give rise to visual field defects. A significant sequela, optic disc pit maculopathy (ODPM), develops in 25%–75% of cases and leads to visual impairment due to the accumulation of intraretinal and subretinal fluid at the macula, accompanied by alterations in the retinal pigment epithelium [2]. Owing to the rarity and poorly understood pathogenesis of ODPM, there is currently no established consensus regarding its optimal management. Nevertheless, recent literature highlights a growing preference for pars plana vitrectomy (PPV) in conjunction with adjunctive procedures, such as the application of an inverted autologous internal limiting membrane (ILM) flap to cover the ODP [3–8]. This report delineates the inverted ILM flap technique for the treatment of ODPM, demonstrating substantial retinal fluid absorption and marked improvement in visual acuity at a 16-month follow-up.

## Case report

**Figure 1:**
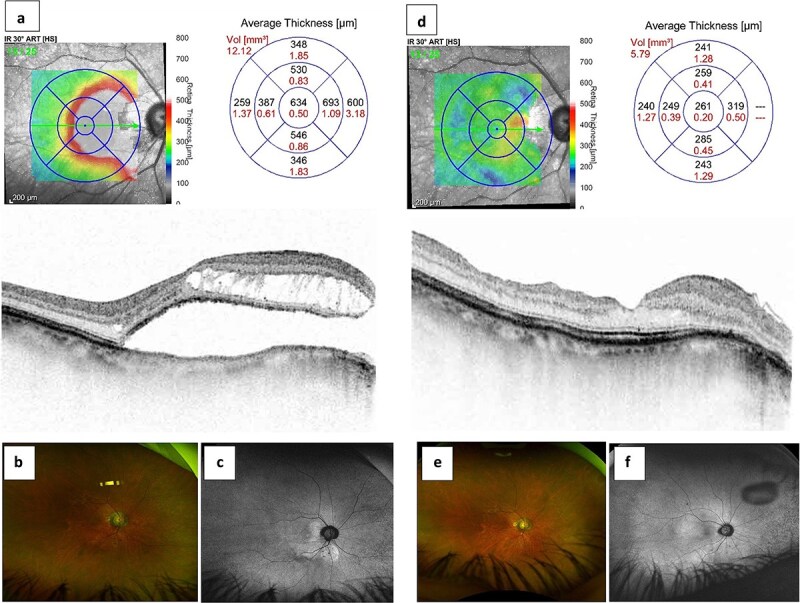
Efficiency of pars plana vitrectomy associated with inverted internal limiting membrane flap technique for optic disc pit maculopathy. The preoperative optical coherence tomography (a) and ultra-widefield scanning laser ophthalmoscopy (b and c) revealed intraretinal and subretinal fluid associated with a macular detachment of central foveal thickness (CFT) of 634 μm in the right eye. The improvement of CFT of 261 μm (d) with complete resolution of intraretinal and subretinal fluid (e and f) after surgery.

A 35-year-old woman in overall good health presented with progressive visual impairment in her right eye over the preceding 7 months. Ophthalmologic evaluation revealed a best-corrected visual acuity of 20/100 in the right eye and 20/20 in the left eye. Anterior segment examination was unremarkable bilaterally. Optical coherence tomography (Spectralis OCT, Heidelberg, Germany) of the right eye demonstrated ODPM (Fig. 1a), characterized by the presence of both intraretinal and subretinal fluid, as well as a substantial macular detachment with a central foveal thickness (CFT) of 634 μm. Fundus ultra-widefield scanning laser ophthalmoscopy (Optos, Inc., Nikon, MA, USA) further corroborated these findings (Fig. 1b and c). In contrast, the left eye exhibited a temporal ODP without associated macular changes. Visual field testing revealed an enlarged blind spot in the right eye compared to the left.

Following the acquisition of informed consent, the patient underwent a standard 23-gauge, three-port PPV under retrobulbar anesthesia. Posterior vitreous detachment (PVD) was initially induced, succeeded by meticulous removal of the residual fine premacular posterior cortical vitreous. A 0.125% solution of indocyanine green was applied to the macular region to facilitate visualization, and any excess dye was promptly aspirated.

Subsequently, the ILM was delicately elevated in a circular fashion, encompassing an area ~2.5 times the diameter of the optic disc centered on the macula. Notably, the nasal aspect of the ILM was intentionally left attached to the retina, thereby preserving its connection at the nasal margin of the macula and creating a temporally based ILM flap of approximately two disc diameters. This flap was then inverted nasally using intraocular forceps, effectively covering and filling the ODP. Gentle manipulation ensured the flattened apposition of the inverted ILM flap over the optic disc. The procedure concluded with a fluid-gas exchange utilizing a 25% concentration of sulfur hexafluoride (SF_6_) as an intraocular tamponade. Postoperatively, the patient was instructed to maintain a facedown position for a duration of 3 days.

Over the course of 8 months of follow-up, there was marked absorption of both intraretinal and subretinal fluid, culminating in the complete resolution of CFT to 261 μm (Fig. 1d). Visual acuity in the right eye improved to 20/20, and the visual field blind spot normalized. Postoperative OCT and ultra-widefield imaging confirmed that the inverted and folded ILM flap effectively covered the disc pit, resulting in the sustained resolution of ODPM (Fig. 1e and f) at the 16-month follow-up.

## Discussion

The patient underwent PPV, induction of PVD, ILM peeling to create an ILM flap, inversion of the ILM flap to cover the disc pit, and gas tamponade as a comprehensive approach to address the pathogenesis of maculopathy associated with ODP. The rationale for employing PPV lies in its capacity to eliminate vitreous traction at both the optic disc and macula in cases of ODPM, a factor deemed critical for the resolution of intraretinal and subretinal fluid and subsequent macular reattachment [2].

A fundamental principle in the management of ODPM is the establishment of a permanent barrier to impede the migration of fluid from the ODP. The inverted ILM flap represents a refined surgical technique designed to occlude the ODP, thereby obstructing communication between the pit and the subretinal or intraretinal spaces and preventing further accumulation of fluid within the macula. Recent hospital-based studies have demonstrated that patients with an ILM flap covering the pit exhibit higher rates of complete anatomical success compared to those who underwent ILM peeling alone [5]. The safety and efficacy of the inverted ILM flap technique have been substantiated in patients with varying sizes of full-thickness macular holes, underscoring its versatility [9]. This approach effectively eliminates both anteroposterior and tangential tractional forces, thereby facilitating closure of the communicating channel between the macula and the optic disc.

## Conclusion

In our case, the implementation of the inverted ILM flap technique yielded a marked improvement, as evidenced by OCT demonstrating resolution of both macular schisis and serous retinal detachment, with CFT decreasing from 634 to 261 μm and visual acuity improving from 20/100 to 20/20. These findings are congruent with those reported in various case series, which indicate that PPV combined with inverted ILM flap insertion over the optic disc consistently results in favorable anatomical and visual outcomes in patients with ODPM within 1 year of follow-up [4, 7, 8, 10, 11]. Furthermore, both our observations and those of other investigators [6] have substantiated that the resolution of retinal fluid can be sustained over long-term follow-up. The successful anatomical and functional results achieved in this case further support the growing adoption of PPV with inverted ILM flap as an effective therapeutic modality for ODPM, facilitating both fluid resolution and visual improvement over extended observation periods.

While additional large-scale, comparative studies are warranted to definitively establish the optimal surgical approach, this case report contributes valuable evidence supporting the benefits of this technique in the management of serous macular detachment secondary to ODP. By addressing underlying tractional forces and occluding the communication between the pit and the macula, PPV in conjunction with the inverted ILM flap substantially enhances the likelihood of visual recovery and promotes long-term retinal stability.
